# Atopic dermatitis in Ethiopian children: a multicenter study of clinical severity, characteristics, and sociodemographic factors

**DOI:** 10.3389/fmed.2024.1410310

**Published:** 2024-07-15

**Authors:** Abraham Getachew Kelbore, Wendemagegn Enbiale, Jacqueline M. van Wyk, Anisa Mosam

**Affiliations:** ^1^Department of Dermatology, College of Health Sciences and Medicine, Wolaita Sodo University, Wolaita Sodo, Ethiopia; ^2^Department of Dermatology, Nelson R Mandela School of Medicine, University of KwaZulu-Natal, Durban, South Africa; ^3^Department of Dermatology, College of Health Sciences and Medicine, Bahir Dar University, Bahir Dar, Ethiopia; ^4^Amsterdam UMC, University of Amsterdam, Department of Dermatology, Amsterdam Institute for Infection and Immunity (AII), Location Academic Medical Centre, Amsterdam, Netherlands; ^5^Nelson R. Mandela School of Medicine, University of KwaZulu-Natal, Durban, South Africa; ^6^Department of Health Sciences Education, University of Cape Town, Cape Town, South Africa; ^7^Inkosi Albert Luthuli Central Hospital, Durban, South Africa

**Keywords:** atopic eczema, pediatrics, SCORAD index, demographic factors, hospital, Ethiopia

## Abstract

**Background:**

Atopic dermatitis (AD) is a chronic relapsing, pruritic, inflammatory skin disease. Assessing the characteristics and risk factors of severe AD is central to healthcare workers’ understanding and subsequent education of patients for the most optimal outcomes. The clinical characteristics are known to vary depending on populations and regions. AD has been well-documented in the global North in mainly Caucasian populations, while very few studies have been conducted on African patients residing in Africa. This study assessed the clinical characteristics, severity, and sociodemographic factors of children with AD in Southern Ethiopia.

**Methods:**

A hospital-based cross-sectional study was conducted among 461 children and their caregivers in four randomly selected hospitals in Southern Ethiopia from October 2022 to September 2023. A systematic sampling technique was used to enroll study participants. Clinical profile and sociodemographic data were collected by trained data collectors. The Scoring Atopic Dermatitis (SCORAD) index tool was used. The descriptive analysis was performed to characterize study participants. Univariate and ordinary logistic regression were used to identify factors associated with the SCORAD index score. The OR with 95% was used to show the strength of association, and a *p-*value of <0.05 was used to declare the level of significance.

**Result:**

Out of 461 AD-diagnosed children, 212 (46%) were girls and 249 (54%) were boys. In the sample of pediatric patients, 149 (32.3%) exhibited mild AD, 231 (46.2%) presented with moderate, and 99 (21.5%) showed signs and symptoms of severe AD. All patients had itching. Dryness of skin, excoriation, and erythema, followed by lichenification, were the most observed signs. In the ordinary logistic regression model, age onset of the disease [AOR 95% CI 1.95 (1.3–2.94)], sex of caregiver or family [AOR 95% CI 0.61 (0.41–0.90)], family atopy history [AOR 95% CI 0.64 (0.44–0.93)], mother education status [95% CI 2.45 (1.1–5.47)], and use of herbal medication [AOR 95% CI 0.50 (0.33–0.79)] were significantly associated with the severity of AD.

**Conclusion:**

In this study, 68% of children were found to have moderate-to-severe AD. Early onset, maternal education, familial atopy history, sex of caregiver, and use of herbal medication were independent predictors of severe AD in children. We recommend further investigation into these variables for their potential to serve as markers to assess the severity of AD and improve the care and management of children with AD in Ethiopia.

## Introduction

Atopic dermatitis (AD) is a chronic, intensely itchy inflammatory skin disease that occurs most frequently in children but can also affect adults. It is a debilitating disease that affects up to 25% of children and 3% of adults worldwide (1). The onset is most commonly noted from the age of 3–6 months, with nearly 60% of children developing AD in their first year and 90% of patients by the age of 5 (2).

AD is a multifactorial disease, and environmental factors have been found to play a role in individuals who are genetically predisposed to the disease (3). Heredity is an important biological risk factor in the occurrence of immune sensitization and allergy (4). Clinically, AD can present in three forms based on age and morphologic distribution, i.e., infantile, childhood, and adulthood atopic dermatitis (5). The clinical characteristics and sociodemographics of AD have been thoroughly documented in developed nations, and these characteristics are applied globally. Flexural dermatitis, the classic presentation of AD, is more likely to develop in European American skin than in African American skin. Conversely, extensor lesions are more prevalent in AD patients from South Africa (6) and Nigeria (7), particularly in younger individuals. Despite differences in characteristics among populations, it is important to characterize AD in Ethiopian skin.

AD is diagnosed clinically. There are different diagnostic criteria for use in clinical practice and research. The UK Working Party’s Diagnostic Criteria and Hanifin and Rajka criteria are the two most commonly used criteria (8, 9). However, we used a validated UK Working Party’s Diagnostic Criteria tool for Ethiopian children (10). In Ethiopia, AD is the most frequently diagnosed inflammatory skin disease among children who visit public hospitals (11, 12). The standard treatment of AD includes emollients, topical corticosteroids, education on avoidance of eliciting factors, and the treatment of infections, which are the main pillars of treatment (13).

There is no clear laboratory test that helps to identify the severity of AD; however, classifying the severity of the disease is important to direct therapy. The most common and widely used tool to assess the severity of AD is the SCORAD (Scoring of Atopic Dermatitis) index (14). The tool includes seven objective parameters and two subjective parameters, which are suitable for clinical research but complicated for everyday practice (15). Age at early onset of AD, sex, family atopy history, rural residence, passive cigarette smoke exposure and filaggrin mutation (16), severe and persistent disease, and allergic sensitization were reported as predictors for the clinical severity of AD among children (17, 18). Knowledge of factors that determine disease severity in patients with AD is essential to inform and educate about preventive measures to optimize care for children with AD. However, there is a lack of evidence of factors associated with the severity of atopic dermatitis among children in the Ethiopian context. This study was thus conducted to investigate the clinical characteristics and associated factors of severity of atopic dermatitis among children in Southern Ethiopia.

## Methods and materials

### General setting

This study was conducted in the Southern Nations, Nationalities, and Peoples Regional State (SNNPRS). The Southern Nations, Nationalities, and Peoples Region (SNNPR) is the third largest administrative region in Ethiopia and is the most diverse region in terms of languages, cultures, and ethnic backgrounds. Administratively, the region is divided into 16 zones (sub-provinces), one city administration, and seven special “woredas” or districts. The region has an estimated population of 21,492,925 in 2022. The region has cold, temperate, and dry weather conditions, and the majority of the region’s population has been dependent on un-mechanized agriculture and domestic animal raring, whereas small businesses are also becoming part of livelihood practice these days (19).

The Annual Regional Health Bureau 2021/2022 report describes the area as having a total of 76 governmental hospitals, 723 health centers, and 3,874 health posts (20). Throughout the country, there are less than 180 dermatology professionals, and the public healthcare facilities are organized into a three-tier healthcare system called primary (primary healthcare units), secondary, and tertiary levels. The primary healthcare unit consists of the primary hospitals, health centers, and health posts. The primary hospitals serve as referral centers for their catchment health centers and deliver some emergency surgical care and curative services; however, there is no primary hospital and health center that provides dermatology services in the region. The health center supervises up to five satellite health posts, and it is the entry point for many curative services for up to 40,000 in urban and 25,000 people in rural areas. The health post is a community-level public healthcare level that mainly delivers preventive care for up to 500 households. The secondary level stands for general hospitals where some advanced services, including dermatology services, are being given for its catchment population; nonetheless, only 4 of the 10 general hospitals have dermatology services, and the tertiary level stands for referral and teaching hospitals where the most advanced healthcare including dermatology services are being delivered.

In general, only nine hospitals provide dermatology services, with a total of 14 dermatology professionals; the overall dermatology services are neither accessible nor available in most of the health facilities, and they do not have adequately trained dermatology professionals to diagnose atopic dermatitis (20–22).

### Specific setting, study design, and period

This study is a cross-sectional part of a large prospective cohort study among children with atopic dermatitis. The study was conducted in four public hospitals in southern Ethiopia, which include two comprehensive specialized hospitals (Wolaita Sodo University at Sodo and Wachamo university Nigist Eleni Mohammad memorial at Hossana) and two general hospitals (Arbaminch from Gamo zone and Dr. Bogalech Gebre Memorial General Hospital from Kembata zone). Selected hospitals provide holistic dermatology services both through outpatient and inpatient units of the dermatology department and are managed by dermatology professionals. The study was conducted from October 2022 to September 2023.

### Study population

The population included all children 16 years of age and younger who have been diagnosed with atopic dermatitis and patients’ families or caregivers during the study period.

Patient’s inclusion and exclusion criteria: Children or pediatric patients diagnosed with atopic dermatitis during the study period are eligible. Children with a known psychiatric problem, a history of steroid and antihistamine treatment intake within the last 2 weeks, and families or caregivers aged 18 years or less were excluded.

### Sample size determination

The sample size required for the study was calculated using the formula to estimate a single population proportion.


$$n=\frac{z^{2}p\left(1-p\right)}{d^{2}}$$

Where *n* = desired sample size.

*Z* = level of significance at 95% confidence interval.

*p* = proportion of atopic dermatitis among children (11.3%) (12).

*d* = margin of error (0.03%)


$$\frac{1.96^{2}*0.113\left(1-0.113\right)}{0.03^{2}}=428$$

After adding a 10% non-response rate, the final sample size was 470. Accordingly, the total sample size of the study was 470.

### Sampling technique

The SNNPR of Ethiopia consists of 16 zones and 7 special districts but all 7 special districts and nine zones do not have public health facilities that provide dermatology services for the people. From the remaining seven zones that have dermatology services, four were randomly selected. Thereafter, the available hospital that offers dermatological services from the selected zones were purposively chosen, and the sample size was proportionally allocated to each hospital based on their last 6 months’ average number of patients visiting each hospital. Then, the hospitals were stratified into the ranks of the hospitals (i.e., secondary and tertiary levels). Finally, participants, parents, or caregivers from the selected hospital were selected through systematic sampling. Accordingly, 464 study participants were included in the study.

### Study variables and measurement

#### Variables

The main research variables are clinical profile of patients with atopic dermatitis, age, sex, residence, family occupation, family educational status, family monthly income, duration, marital status of caregiver/family, blood relation with caregiver, atopy history, onset of age, duration since diagnosed, comorbidity, sex of caregiver/family, history of smoking person in the family, support for AD children, herbal medication use, housing type, and clinical severity of the disease according to the SCORAD index.

### Stating diagnostic criteria

In this study, the UK Working Party’s Diagnostic Criteria for atopic dermatitis were used to ascertain the diagnosis, and it is a validated tool for Ethiopian children (8, 10). The minimum criteria for diagnosis include an itchy skin condition plus three or more of the following: a history of flexural involvement, a history of personal or family atopy, a history of generalized dry skin, the onset before the age of 2 years, or visible flexural dermatitis.

#### Measurement

Dermatology clinicians assessed the severity using the SCORAD index, which considers both objective and subjective indicators of AD. The clinical features used to assess disease severity are erythema, swelling, oozing/crusting, excoriation, lichenification, and dryness. Each characteristic is rated with 0, 1, 2, or 3, depending on the severity. The extent of diseases is calculated according to the percentage of body surface area affected with eczema by using the rule of nine as per the age of a child.

The SCORAD assesses subjective symptoms, that is, the intensity of itch and sleeplessness, on a scale from 0 (no symptom) to 10 (maximum) and refers to the previous 3 days. The numerical data on all three aspects of the disease are entered into tables and summed, with a maximum score of 103. All items were completed on a SCORAD evaluation form. Individual item scores were then combined according to the following formula: A/5 + 7B/2 + C, where A stands for extent, B for intensity, and C for symptoms. A SCORAD index result of less than 25 indicated mild disease, 25 to 50 as moderate disease severity, and greater than 50 as severe AD. Asymptomatic patients have a SCORAD result of 0.

### Operational definition

A caregiver is someone who may or may not have a blood relationship with the child but provides care for the sick child at home and anywhere else.

Family support is defined as specific atopic dermatitis skin care and/or attention given to the child because of the AD from the family member(s).

### Data collection tool

A structured questionnaire was developed based on the review of literature and adapted to fit the regional context of the study area (8, 14, 17, 18, 23, 24). All the tools were translated from English to Amharic and then back to English by different professional translators to ensure the consistency of the information.

Then, the tool was pre-tested on 5% of the sample at the Wolaita Sodo Christian Hospital pediatric outpatient unit, which was not part of the study site. Dermatology professionals examined the patients, and data collectors (nurses) were trained on the data collection tool and procedures and getting informed consent before starting the data collection. The data collection was carried out at the dermatology outpatient unit in each hospital.

#### Data processing and analysis

Collected data were double-entered from the paper-based data collection sheets into Epi-Data software (*v4.2.0.0 for entry Epi-Data Association, Odense, Denmark*). The data were cleaned by running frequency and cross-tabulation using Epi-data and then exported to SPSS version 27 for analysis. Descriptive statistical methods, such as frequencies, the proportion at 95% confidence interval (CI), mean, standard deviation, and median, were used. The occurrence of multicollinearity was checked among candidate variables using tolerance and variance inflation factor (VIF) and proved it has no multicollinearity. Model fitness was checked before the final analysis, and the model was found to be fit (*p*-value 0.52). Ordinary logistic regression was used to identify the predicting variables for the outcome variable(s). All variables were analyzed in bivariable logistic regression, and those variables with a *p*-value less than 0.25 were entered into multivariable logistic regression analyses. Statistical significance was declared at a *p-*value of <0.05.

### Data quality assurance

To maintain the quality of data, a pre-validated standard questionnaire was used for data collection. Continuous supervision was done during data collection at each hospital, and 10% of the dataset was double-entered to check the accuracy of the entered data.

## Results

### Sociodemographic characteristics

A total of 461 atopic dermatitis-diagnosed children and their caregivers or families (461) were included with a response rate of 98.1%. Two hundred and forty-nine (54.01%) of the children were boys. The age of the participants ranged from 6 months to 16 years (the median age of children was 3 years and the interquartile range (IQR) was 1,5), and the largest proportion of children was less than 5 years old (312, 67.7%). The age of caregivers ranged from 18 to 54 years (mean 30.43 ± 5.84 years). A total of 315 (68.3%) caregivers were women and 408 (88.5%) caregivers were married. A total of 287 (62.3%) participants in this study resided in urban areas; 185 (40.1%) mothers and 220 (47.7%) fathers had a tertiary educational level (Table 1).

**Table 1:** The sociodemographic and family status among atopic dermatitis-diagnosed children and their families in Southern Ethiopia, 2023.

Variable	Category	Frequency	Percent
Sex	Female	212	46
	Male	249	54
Age of children	≤4 years	312	67.7
	5–10 years	102	22.1
	11–16 years	47	10.2
Residence	Rural	174	37.7
	Urban	287	62.3
Family occupation	Employee	185	40.1
	Farmer	59	12.8
	Housewife	77	17.7
	Merchant	140	30.4
Monthly income in ETB	≤5000ETB	224	48.6
	5,001–10000ETB	178	38.6
	≥10001ETB	59	12.8
Education status mother	No formal education	102	22.1
	Primary school	99	21.5
	Secondary school	75	16.3
	Tertiary (Diploma and above)	185	40.1
Education status father	No formal education	79	17.1
	Primary school	82	17.8
	Secondary school	80	17.4
	Tertiary (Diploma and above)	220	47.7
Marital status caregiver	Widowed/divorced	23	5
	Married	408	88.5
	Single	30	6.5
Age of caregiver	18–24 years	41	8.9
	25–34 years	295	64
	≥35 years	125	27.1
Caregiver sex	Female	315	68.3
	Male	146	31.7
Type of housing	Private housing condominium	35	7.6
	Public housing 1–3 rooms	132	28.6
	Public housing 4–5 rooms	43	9.3
	Private housing villa or landed	251	54.5
Number of children	≤ 2	238	51.6
	3–4	146	31.7
	≥5	77	16.7

### Clinical characteristics of atopic dermatitis diagnosed children

In this study, the initial appearance of signs and symptoms on the skin was noticed in 370 (80.3%) of the children before the age of 2, and the common clinical features observed among AD children were dryness of skin (xerosis) (419, 90.9%), erythema or redness (392, 85%), excoriation/scratch marks (366, 79.4%), lichenification (332, 72%), edema/swelling (287, 62.3%), and oozing/crust (245, 53.1%). A history of itching was reported in all AD-diagnosed patients with different levels of extent. The mean of body surface involvement was 34.5 ± 20.6, with a range of 8 to 100%. The distribution of the body parts affected were the head (that includes face and scalp) (356, 77.2%), upper limbs involving fossa cubitalis (345, 74.8%), and lower limb (310, 67.2%), followed by the trunk involvement (210, 45.6%) and front neck 154 (33.4%). The distribution of head and face involvement was higher among children aged younger than 4 years (300, 84.3%), followed by 5- to 10-year-olds (38, 10.7%) and 11- to 16-year-olds (18, 5%).

Sixty-one percent of children had a family atopy history, with mother 141 (30.6%), father 77 (16.7%), siblings 28 (6%), and grandparents 35 (7.6%) reporting a relationship with AD children. A history of passive smoking (i.e., where someone in the household smoked that was inhaled by the child) was recorded in 63 (13.67%) of the pediatric patients. One hundred forty-two (30.8%) of the children had a history of herbal medication use for AD treatment.

Family support was reported for 79 (17.1%) AD-affected children and 99 (21.5%) of the children had other comorbidities. The most reported comorbidity was asthma, affecting 71 (71.2%) of these children (Table 2).

**Table 2:** Clinical characteristics profile among atopic dermatitis-diagnosed children and their families in Southern Ethiopia, 2023.

Variables	Category	Frequency (*N*)	Percent (%)
Age of onset	< 2 years	370	80.3
	≥ 2 years	91	19.7
How long since diagnosed	< 1 year	310	67.2
	≥ 1 year	151	32.8
Blood relation with caregiver	Mother	286	62
	Father	125	27.1
	Siblings	26	5.6
	Other	24	5.2
Smoking person in family	No	398	86.3
	Yes	63	13.7
Herbal medication use	No	319	69.2
	Yes	142	30.8
Family atopy history	No	180	39
	Yes	281	61
Probands/relationship	Mother	141	30.6
	Father	77	16.7
	Siblings	28	6.1
	Grandparent	35	7.6
Family support	No	382	82.9
	Yes	79	17.1
Comorbidity of child	No	362	78.5
	Yes	99	21.5
Comorbid diseases of child	Asthma	71	71.7
	Diabetes mellitus	19	19.2
	Other	9	9.1
SCORAD index	Mild	149	32.3
	Moderate	231	46.2
	Severe	99	21.5

### Severity of atopic dermatitis and its distribution across age, sex, and onset of the disease

The mean SCORAD index for the patients in this study was 37.15 ± 18.42. According to the SCORAD severity scale, 149 (32.3%) patients had mild, 231 AD (46.2%) had moderate AD, and 99 (21.5%) had severe AD. Based on the age category of the children, the severity SCORAD index score result ratio increased from mild to severe as age increased; approximately 101 (32.4%) children with age ≤ 4 years had mild AD, 145 (46.5%) children with ≤4 years had moderate AD, and 66 (21.2%) children with ≤4 years had severe AD; approximately 32 (31.4%) children with age 5–10 years had mild AD, 49 (48%) children with age 5–10 years had moderate AD, and 21 (20.6%) children with 5–10 years had severe AD, and 16 (34%), 19 (40.4%), and 12 (25.5%) children with age 11–16 years had mild, moderate, and severe AD, respectively.

Nearly 40% of children in this study had moderate-to-severe AD, and they lived in a rural location. Among children with a history of familial atopy, 84 (29.9%) were identified with mild AD, 124 (44.1%) with moderate AD, and 73 (26%) with severe AD. The distribution of AD severity according to the onset of age is different and more common among children with age less than 2 years (335, 72.7%); of these, 115 (34.3%) had mild AD, 168 (50.1%) had moderate AD, and 52 (15.5%) had severe AD (Table 2).

### Factors associated with the severity of atopic dermatitis according to the SCORAD index measurement

In univariate logistic regression, age onset, residence, family occupation, maternal and parental education status, family atopy history, history of herbal medication use, sex of caregiver or family, child comorbidity, and the number of children/siblings were associated with the severity of AD according to the SCORAD index.

The SCORAD index measurement and ordinary regression analysis revealed factors that were significantly associated with the severity of AD. These factors included early age onset of the disease, sex of caregiver, family atopy history, mother’s educational status, and use of herbal medication.

The odds of developing severe AD are two times (AOR, 1.95, 95% CI,1.3, 2.94) higher among children whose onset of age is less than 2 years when compared to children who develop AD after 2 years.

Children whose maternal educational level was at the secondary level had approximately three times (AOR, 2.77, 95% CI 1.26, 6.1) higher odds of developing AD, and those with mothers with tertiary qualifications had 2.5 times (AOR, 2.45, 95% CI 1.1, 5.47) higher odds of developing AD compared to children whose mothers did not have a formal education.

The risk of developing severe AD decreased by 36% (AOR, 0.64, 95% CI: 0.44, 0.93) among children with a family history of atopy when compared to children without a family history.

The risk of developing severe AD was 39% (AOR, 0.61, 95% CI 0.41, 0.90) lower among children whose caregivers were women compared to those children whose caregivers were men.

The risk of developing severe AD was reduced by 50% (AOR, 0.50 95% CI 0.33, 0.79) among children who have a history of herbal medication use when compared to those who did not use herbal medication (Table 3).

**Table 3:** Factors associated with the severity of AD according to the SCORAD index measurement among children in Southern Ethiopia 2023.

Variable characteristics	Mild *N* (%)	Moderate *N* (%)	Severe *N* (%)	Univariate COR (95% CI)	Multivariate AOR (95% CI)	*p* value
Age of onset						
< 2 years	115 (34.3)	168 (50.1)	52 (15.5)	2.16 (1.45–3.22)	1.95 (1.3–2.94)	0.001*
≥ 2 years	34 (27)	45 (35.7)	47 (37.3)	1	1	
Residence						
Rural	50 (27)	87 (47)	48 (25.9)	0.66 (0.46–0.93)	0.7 (0.48–1.05)	0.083
Urban	99 (35.9)	126 (45.7)	51 (18.5)	1	1	
Family occupation						
Employee	71 (38.4)	81 (43.8)	33 (17.8)	1.7 (1.1–2.5)	1.11 (0.66–1.86)	0.686
Farmer	19 (32.2)	26 (44.1)	14 (23.7)	1.23 (0.7–2.18)	1.74 (0.89–3.42)	0.108
Housewife	22 (28.6)	39 (50.6)	16 (20.8)	1.2 (0.71–2)	1.43 (0.81–2.51)	0.214
Merchant	37 (26.4)	67 (47.9)	36 (25.7)	1	1	
Mother education						
Tertiary (Diploma and above)	70 (37.8)	86 (46.5)	29 (15.7)	2.8 (1.76–4.46)	2.45 (1.1–5.47)	0.028*
Secondary school	33 (44)	29 (38.7)	13 (17.3)	3.26 (1.83–5.82)	2.77 (1.26–6.1)	0.011*
Primary school	26 (26.3)	53 (53.5)	20 (20.2)	1.82 (1.08–3.07)	1.69 (0.83–3.43)	0.148
No formal education	20 (19.6)	45 (44.1)	37 (36.3)	1	1	
Father education						
Tertiary (Diploma and above)	79 (35.9)	104 (47.3)	37(16.8)	3(1.82–4.98)	0.89 (0.38–2.1)	0.788
Secondary school	35 (43.8)	33 (41.3)	12(15)	3.97 (2.16–7.3)	1.34 (0.57–3.14)	0.503
Primary school	19 (23.2)	46 (56.1)	17(20.7)	1.9 (1.08–3.48)	1.07 (0.50–2.3)	0.868
No formal education	16 (20.3)	30 (38)	33 (41.8)	1	1	
Number of children						
≤ 2	88 (37)	111 (46.6)	39 (16.4)	2.03 (1.24–3.3)	1.44 (0.83–2.50)	0.195
3 to 4	41 (28.1)	70 (47.9)	35 (24)	1.3 (0.78–2.24)	0.94 (0.53–1.65)	0.826
≥5	20 (26)	32 (41.6)	25 (32.5)	1	1	
Family support						
Yes	23 (24.7)	39 (41.9)	31 (33.3)	0.53 (0.34–0.82)	0.80 (0.50–1.29)	0.363
No	126 (34.2)	174 (47.3)	68 (18.5)	1	1	
Family atopy history						
Yes	84 (29.9)	124 (44.1)	73(26)	0.64 (0.45–0.91)	0.64 (0.44–0.93)	0.019*
No	65 (36.1)	89 (49.4)	26 (14.4)	1	1	
Sex of caregiver						
Female	91 (28.9)	147 (46.7)	77 (24.4)	0.59 (0.41–0.86)	0.61 (0.41–0.90)	0.013*
Male	58(39.7)	66(45.2)	22(15.1)	1	1	
Herbal medication use						
Yes	27 (19)	67 (47.2)	48 (33.8)	0.38 (0.26–0.55)	0.50 (0.33–0.79)	0.002*
No	122(38.2)	146(45.8)	51(16)	1	1	
Child comorbidity						
Yes	29 (29.3)	35 (35.4)	35(35.4)	0.57 (037–0.88)	0.74 (0.48–1.16)	0.195
No	120 (33.1)	178 (49.2)	64 (17.7)	1	1	

1, Reference category; *, Statistical significance at *p* < 0.05; Bold values just for showing significantly associated variables *p* < 0.05.

## Discussion

The results from this study showed that more than two-thirds of children had moderate-to-severe AD. The involvement of the head and neck (including the face and scalp) was higher, followed by the upper limbs, lower limbs (67.2%), and trunk, with dryness and itchy skin. Several factors contributed to children being more prone to severe AD. These factors included early age onset of symptoms, family atopy history, history of herbal medication use, sex of the caregiver, and maternal education status.

This study examined the severity of AD in children using the SCORAD index. The SCORAD index has been described as the most reliable and feasible evaluation for AD (14). In this study, more than two-thirds of patients had moderate-to-severe AD (32.3% of patients had mild, 46.2% had moderate, and 21.5% had severe AD). This finding aligns with the consistent findings from studies conducted in Denmark and Turkey (23, 24). Unlike the results in this study, other epidemiological studies reported that approximately 75% of patients with AD have mild disease, 20% have moderate disease, and 5% have severe disease (25–27).

The possible reasons for the differences could be explained by the study sample size, study design, geographical differences, access to dermatology services, and the tools used to measure the severity and clinical experience of AD. Nonetheless, the assessment of disease severity serves as the recommended first step when selecting treatment options (28, 29).

In a hospital-based study from South Africa, over 80% of children had involvement of the head and neck (including the face and scalp) (6). A study from China reported a similarly high proportion (86%) of facial dermatitis (30). In our study, involvement of the head and neck (including the face and scalp) was observed in 78.2% of children, followed by the upper limbs at 74.8%, lower limbs at 67.2%, and trunk at 45.6%. These findings are consistent with the clinical features of African AD reported in a previous meta-analysis (31). Upper and lower extremities (limbs) are more commonly involved than the trunk, as flexural and extensor areas of the limbs are known predilection areas of AD. Dryness of skin (xerosis) (90.9%), erythema or redness of the skin (85%), excoriation/ scratch marks (79.4%), and lichenification (72%) were the common clinical features observed in many children in this study and had been reported as common in children from Africa (32, 33).

The proportion of children with mild-to-severe atopic dermatitis is expected to grow with age (26). Similarly, in this study, we observed that the ratio of mild-to-severe AD increased with the age of children. The proportion of moderate-to-severe AD children was higher in children whose AD onset started before their second birthday. In this study, we identified the early onset of AD before age 2 as an independent predictor that is associated with severe AD development. This result is consistent with that reported in a study conducted in Turkey (18). Early start of AD appears to be associated with a higher total blood IgE level, implying that early onset of AD may lead to increased allergic sensitization, resulting in more severe eczema. As a result, an early onset of AD may result in a more severe condition, allowing for allergy sensitization due to reduced skin barrier function (34). Therefore, cases with early onset AD symptoms should be monitored more closely and treated due to the high possibility of a severe and persistent clinical course.

A family history of atopy is an independent, strongly associated risk factor for the development of AD (18, 24). Nearly 70% of children with AD have a first-degree relative with some atopic disease (35). In this study, 61% of the children’s patients had a positive atopy family history, and the risk of developing severe AD decreased by 36% among children who had a family history of atopy when compared to those who did not (AOR 0.64, 95% CI 0.44, 0.93). This finding is consistent with those recorded in Turkey (18, 23) and inconsistent with a study conducted in Slovenia and Pakistan (36, 37). The difference could be due to recall bias, age group of study population difference, study design, and terminology used to explain family atopy history. A possible logical explanation can be that parents who have atopic diseases, such as AD or other allergic conditions, allergic rhinitis, or bronchial asthma, probably seek medical advice earlier when the disease is relatively mild.

In this study, maternal education status, especially having a secondary school level and above, was significantly associated with the severity of AD in children. The finding of this study is similar to that of a study conducted in Japan; except for the fact that parental education and income were not associated factors in this study. The odds of developing severe AD are higher and significantly associated with children whose mothers have attained secondary education, a diploma, and/or a degree compared to children whose mothers have less formal education. However, this study is inconsistent with a study conducted by Khalid and Sajid in Pakistan (37). The possible reason for this difference could be the sample size difference; educated families may be more vigilant about their child’s skin health and proactive in using different medications. Severities of AD among children were significantly associated with rural residence (18). However, in our study, this association was not observed. The difference could be attributed to the geographic differences in defining the urban and rural locations, levels of family awareness about the disease, and access to dermatology services. For these findings, a possible justification lies in the hygiene hypothesis theory of atopic dermatitis, which posits an inverse relationship between AD and exposure to endotoxin. AD incidence tends to increase in families who are more educated, have higher incomes, are urban residents, and have smaller family sizes (18, 38, 39).

The assessment of disease severity by a caregiver can greatly impact treatment adherence of the patient (40). In this study, we identified a higher proportion of female caregivers among children with AD, and the odds of developing severe AD were reduced by 39% among AD children with female caregivers compared to male caregivers. It has an association with the severity of AD among children (*p*-value 0.013). Unfortunately, we did not find a study that explains the relationship between caregiver sex and the severity of AD among children. The possible reason could be in the context of Ethiopia, most children are receiving care from their mothers, and they are closer to providing care to their children.

According to this study research, approximately 50% of patients who took herbal medications were shown to be at lower risk for developing severe AD. Herbal medications have certain side effects that include hepatotoxicity, pseudoaldosteronism, and interstitial pneumonia that may occasionally arise after treatment in some patients (41, 42). Nonetheless, the majority of commonly used herbal remedies in Ethiopia lack guidelines about appropriate dosage, duration of treatment, and frequency of application. It does not include descriptions of side effects and indications. Due to this absence of guidelines, the majority of children do not take herbal medications for AD, which may temporarily relieve symptoms of the disease but also cause severe allergies. Therefore, by restoring the function of the skin barrier, decreasing inflammation, and lowering allergy sensitization, early and successful therapies for AD may prevent the advancement of atopic march. Emollients and emollients plus are the baseline therapies recommended for all AD severities (43).

However, studies suggest that herbal medications have anti-inflammatory and immunomodulatory effects (44, 45), but they might not be effective in AD management and prevention of disease severity (46).

This study was one of the few studies that investigated children with AD in Southern Ethiopia. All the children were assessed by dermatology clinicians using the same standard tools. Because of the facility-based cross-sectional nature of the study, our findings on investigating possible factors associated with the increased severity of AD were limited. Particularly, the knowledge about AD and the cultural background of the patients’ caregivers may influence factors such as herbal medication use, caregiver, or family, that we consider in assessing the severity of AD, could not be evaluated within the scope of our study. Further studies are still needed to clarify the factors that may affect the severity of AD in the pediatric population.

## Conclusion

Our findings show that approximately 68% of children were found to have moderate-to-severe AD, which is consistent with studies conducted so far. Early-onset before the age of 2, maternal education, familial atopy history, sex of caregiver, and history of herbal medication use were independent predictors of the severity of AD in children. Since these variables are significantly associated with the SCORAD index, we can postulate that these variables can be used as markers to assess the severity of AD in children to allow optimal care and better management of the cases.

## Data availability statement

The raw data supporting the conclusions of this article will be made available by the authors, without undue reservation.

## Ethics statement

Ethical clearance was obtained from the University of KwaZulu-Natal, Biomedical Research Ethics Committee (BREC) with reference number BREC/00004506/2022. Study permission and gatekeeper’s permission were secured from the Regional Ethiopian Public Health Regional Institute Research and Technology transfer bureau of SNNPRS. Informed written consent was obtained from each participant, patient, parent, or caregiver after providing an explanation of the purpose, benefits, and risks of the study and their right to participate or refuse participation in the study. The anonymity and confidentiality were secured by omitting the participant’s name in any of the questionnaires during data entry, and an identification code only known to the researcher was assigned.

## Author contributions

AGK: Conceptualization, Data curation, Formal analysis, Investigation, Methodology, Project administration, Software, Supervision, Validation, Visualization, Writing – original draft, Writing – review & editing. WE: Conceptualization, Data curation, Investigation, Methodology, Supervision, Validation, Writing – review & editing. JW: Data curation, Methodology, Supervision, Validation, Writing – review & editing. AM: Conceptualization, Data curation, Investigation, Methodology, Supervision, Validation, Writing – review & editing.
